# Discovery of small molecules that normalize the transcriptome and enhance cysteine cathepsin activity in progranulin-deficient microglia

**DOI:** 10.1038/s41598-020-70534-9

**Published:** 2020-08-13

**Authors:** Maria A. Telpoukhovskaia, Kai Liu, Faten A. Sayed, Jon Iker Etchegaray, Min Xie, Lihong Zhan, Yaqiao Li, Yungui Zhou, David Le, Ben A. Bahr, Matthew Bogyo, Sheng Ding, Li Gan

**Affiliations:** 1 Gladstone Institute of Neurological Disease, San Francisco, CA 94158 USA; 2 Department of Neurology, University of California, San Francisco, CA 94158 USA; 3 Gladstone Institute of Cardiovascular Disease, San Francisco, CA 94158 USA; 4 Neuroscience Graduate Program, University of California, San Francisco, CA 94158 USA; 5 Biotechnology Research and Training Center, University of North Carolina At Pembroke, Pembroke, NC 28372 USA; 6 Department of Pathology, Stanford University, Stanford, CA 94305 USA; 7 Helen and Robert Appel Alzheimer’s Disease Research Institute, Brain and Mind Research Institute, Weill Cornell Medicine, New York, NY 10021 USA

**Keywords:** Drug screening, High-throughput screening, Drug discovery, Neuroscience, Neurodegenerative diseases

## Abstract

Patients with frontotemporal dementia (FTD) resulting from granulin (*GRN)* haploinsufficiency have reduced levels of progranulin and exhibit dysregulation in inflammatory and lysosomal networks. Microglia produce high levels of progranulin, and reduction of progranulin in microglia alone is sufficient to recapitulate inflammation, lysosomal dysfunction, and hyperproliferation in a cell-autonomous manner. Therefore, targeting microglial dysfunction caused by progranulin insufficiency represents a potential therapeutic strategy to manage neurodegeneration in FTD. Limitations of current progranulin-enhancing strategies necessitate the discovery of new targets. To identify compounds that can reverse microglial defects in *Grn*-deficient mouse microglia, we performed a compound screen coupled with high throughput sequencing to assess key transcriptional changes in inflammatory and lysosomal pathways. Positive hits from this initial screen were then further narrowed down based on their ability to rescue cathepsin activity, a critical biochemical readout of lysosomal capacity. The screen identified nor-binaltorphimine dihydrochloride (*nor*-BNI) and dibutyryl-cAMP, sodium salt (DB-cAMP) as two phenotypic modulators of progranulin deficiency. In addition, *nor*-BNI and DB-cAMP also rescued cell cycle abnormalities in progranulin-deficient cells. These data highlight the potential of a transcription-based platform for drug screening, and advance two novel lead compounds for FTD.

## Introduction

Progranulin, encoded by the *GRN* gene, is a secreted protein expressed in neurons and microglia in human^1^ and mouse brains^2^. Heterozygous *GRN* mutations lead to progranulin haploinsufficiency and result in frontotemporal dementia (FTD-GRN)^1,3^, a fatal neurodegenerative disease that initially affects behavior and language, with the typical onset from mid 40s to mid 60s^4^. There are currently no treatments available for patients with FTD.

Progranulin’s roles in maintaining lysosomal and inflammatory homeostasis have emerged as key pathogenic drivers of FTD-GRN. Analysis of postmortem brains of patients with *GRN* haploinsufficiency revealed lysosomal dysfunction, evident by increased accumulation of lipofuscin^5^, undegradable lysosomal material that damages cells^6^. In addition, FTD-GRN patients have altered levels of pro-inflammatory cytokines in serum and cerebrospinal fluid (CSF), such as IL-6, although whether this differentiates FTD-GRN from other causes of FTD is unclear^7,8^. In a smaller study, CSF analysis revealed increased levels of IP-10 and decreased levels of IL-15 and TNFα in FTD-GRN patients compared to sporadic FTD cases and healthy controls^9^, further confirming that FTD-GRN patients exhibit an altered inflammatory state.

Conditional knock-out of *Grn* in microglia induces obsessive–compulsive behavior in mice^10^, a key behavioral deficit in FTD-GRN, demonstrating the potential importance of microglial progranulin in this disease. In fact, microglia play a significant role in the etiology and pathology of neurological diseases^11^. Recently, the binary classification of microglia as being in an activated or resting state was challenged^12^. Microglia are now understood to assume several distinct states during repopulation^13^ and disease progression^14^, and a distinct signature of disease-associated microglia (DAM) has been defined^15^. Thus, a technique that can track multiple genes is required to capture the complex state of microglia in a particular disease setting. One such approach is RNA-mediated oligonucleotide annealing, selection, and ligation with next-generation sequencing (RASL-seq), in which a pool of custom designed probes captures target mRNA prey and converts them to amplicons that can then be quantified in parallel through next-generation sequencing^16–18^. RASL-seq is particularly suitable for studying microglia because it allows transcription data for a select number of genes to be obtained for hundreds of conditions in a cost-effective way.

Several therapeutic options are being explored to overcome progranulin haploinsufficiency^19^. A major strategy is to directly enhance progranulin expression level using gene therapy vectors introduced into the medial prefrontal cortex (mPFC)^20,21^, although one study showed that overexpression of *GRN* in *Grn* KO mice led to neurodegeneration^22^. Increased progranulin level can also be achieved by enhancing global transcriptional activity using histone deacetylase (HDAC) inhibitors^23,24^ or by activating autophagy with trehalose^25^. Importantly, neither of these small molecule approaches are specific for *Grn* regulation, and thus may have off-target effects. In addition, achieving the optimal level of progranulin is of paramount importance, since excess progranulin can have carcinogenic effects in some cell types^26^.

To overcome the limitations of progranulin-enhancing strategies, we aimed to develop a new strategy for FTD therapy that does not rely on endogenous *Grn* mRNA expression. Instead, we sought to discover compounds that act as *progranulin activity mimetics* – agents that can mimic progranulin function by partially restoring the transcriptional changes caused by progranulin insufficiency. We utilized a RASL-seq approach to screen a library of bioactive compounds to identify hits that shift the signature profile of *Grn* KO microglia toward the wild-type (WT) state. Because decreased progranulin also impairs lysosomal function^5^, we performed a second, phenotypic screen and discovered two compounds that restored cysteine cathepsin activity in *Grn* KO primary microglia. RNA-seq analysis demonstrated that these compounds also normalized the cell cycle network in *Grn* KO microglia. Overall, we show the feasibility of using RASL-seq technology to discover compounds that could replace important aspects of progranulin’s function in brain cells, and illustrate that compounds that improve lysosomal function in progranulin-deficient microglia can also normalize a major dysregulated transcriptional network.

## Results

### RASL-seq assay development: defining the transcriptomic profile of *Grn* KO and *Grn* WT primary mouse microglia

**Figure 1:**
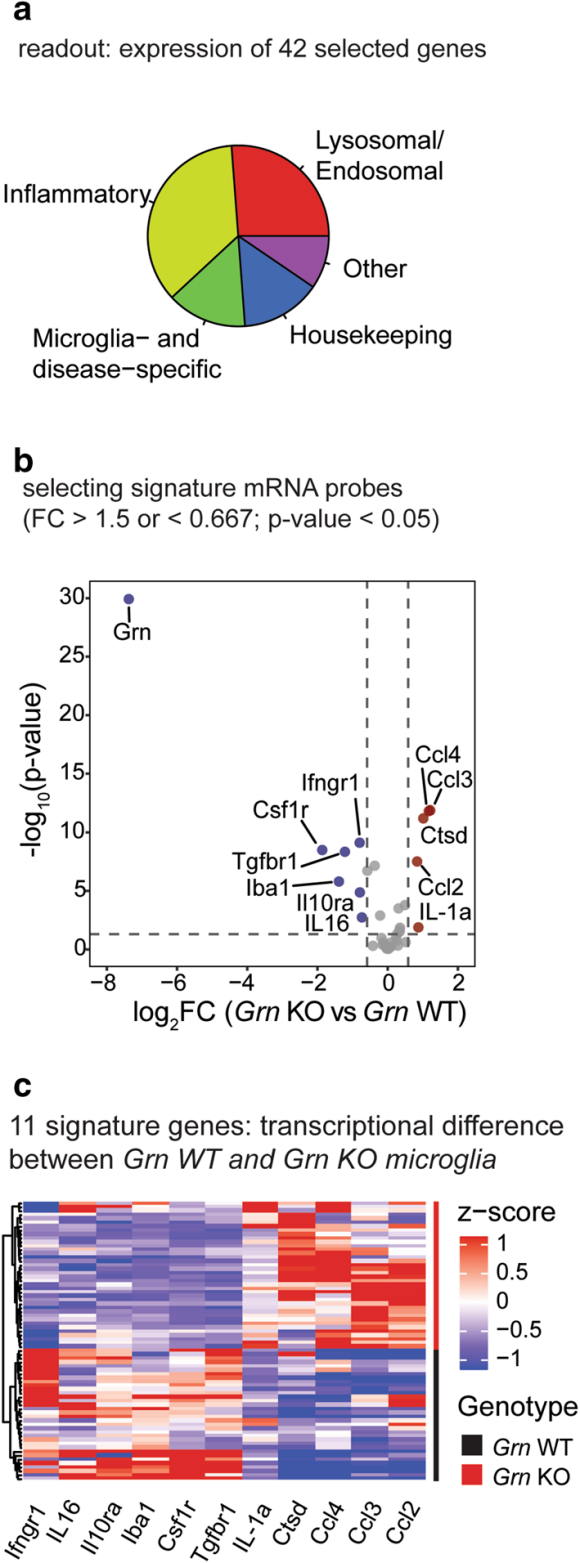
RASL-seq assay development: defining transcriptomic profile for *Grn* KO and *Grn* WT primary mouse microglia. (**a**) Experimental design: RASL-seq gene selection classification. (**b**) Volcano plot identifying signature genes (FC > 1.5 or < 0.667; *p* value < 0.05). (**c**) Heatmap for 11 signature genes differentiating *Grn* WT and *Grn* KO primary mouse microglia with unbiased clustering (Ward’s method with Euclidian distance) into two groups after removal of one outlier.

We first set out to establish the transcriptomic signatures of progranulin insufficient microglia and WT control microglia. We designed a panel of 42 mRNA probes for genes in the lysosomal, inflammatory, and microglial homeostatic pathways, as well as housekeeping genes for normalization (Fig. 1a; Table S1). Several of these genes are part of the “microglial sensome”: microglia-enriched genes that are involved in sensing the brain’s environment^27^. *Grn* WT and *Grn* KO primary mouse microglia were plated in 384-well plates, lysed, and mixed with the 42 mRNA probes. After the RASL procedure and next-generation sequencing, the normalized counts for each targeted transcript were analyzed.

We defined differentially expressed genes between *Grn* KO and WT microglia by a fold change of > 1.5 and *p* value of < 0.05 (Fig. 1b). As expected, the gene with the highest observed transcriptional difference was *Grn*. Of the other 11 signature genes, most were related to inflammation. *Ifngr1*, *IL16*, and *Il10ra* were downregulated in *Grn* KO, while *Il-1α*, *Ccl4, Ccl3*, and *Ccl2* were upregulated, demonstrating a shift instead of a disruption in the inflammatory pathway. Three microglial homeostatic genes, *Iba1*, *Csf1r*, and *Tgfbr1,* were downregulated in *Grn* KO microglia. On the other hand, cathepsin D (*Ctsd*), a lysosomal gene, was upregulated in *Grn* KO compared to WT. Notably, these 11 gene probes demonstrated the most consistent expression patterns across each condition. Therefore, these genes were used as *Grn* KO signature genes for downstream analyses (Fig. 1c).

### Compound screen with RASL-seq identifies compounds that shift transcriptional profiles of *Grn* KO microglia toward the *Grn* WT profile

Next, to identify small molecules that can shift the transcriptional profile of *Grn* KO cells toward WT cells, we screened the Tocris library of 1,120 bioactive compounds with known biological functions (e.g. enzyme inhibitors, ion channel modulators, and receptor agonists and antagonists). *Grn* KO cells were incubated with the compounds, then lysed and profiled with the RASL-seq probes and next-generation sequencing. Wells with less than 40% cell counts compared to the average of non-treated cells and a low number of mRNA reads were filtered out from further analysis, resulting in the removal of 124 compounds. Many of the remaining compounds changed transcriptional profiles of the *Grn* KO cells, as visualized by principal component analysis (PCA) (Fig. S1).

In order to unbiasedly rank the compounds by their effectiveness in normalizing the transcriptional profiles of *Grn* KO cells toward the *Grn* WT cell profile, we gave a score to each compound using Euclidian distance calculation. For this data set, the normalized counts in 11 dimensions of signature genes for all compounds were compared to the mean of the *Grn* WT cells, and a single score corresponding to the Euclidian distance for each compound was calculated for all compound-containing *Grn* KO cells. The smaller the score, the closer the compound-treated *Grn* KO transcriptomic profile is to the WT signature.

**Figure 2:**
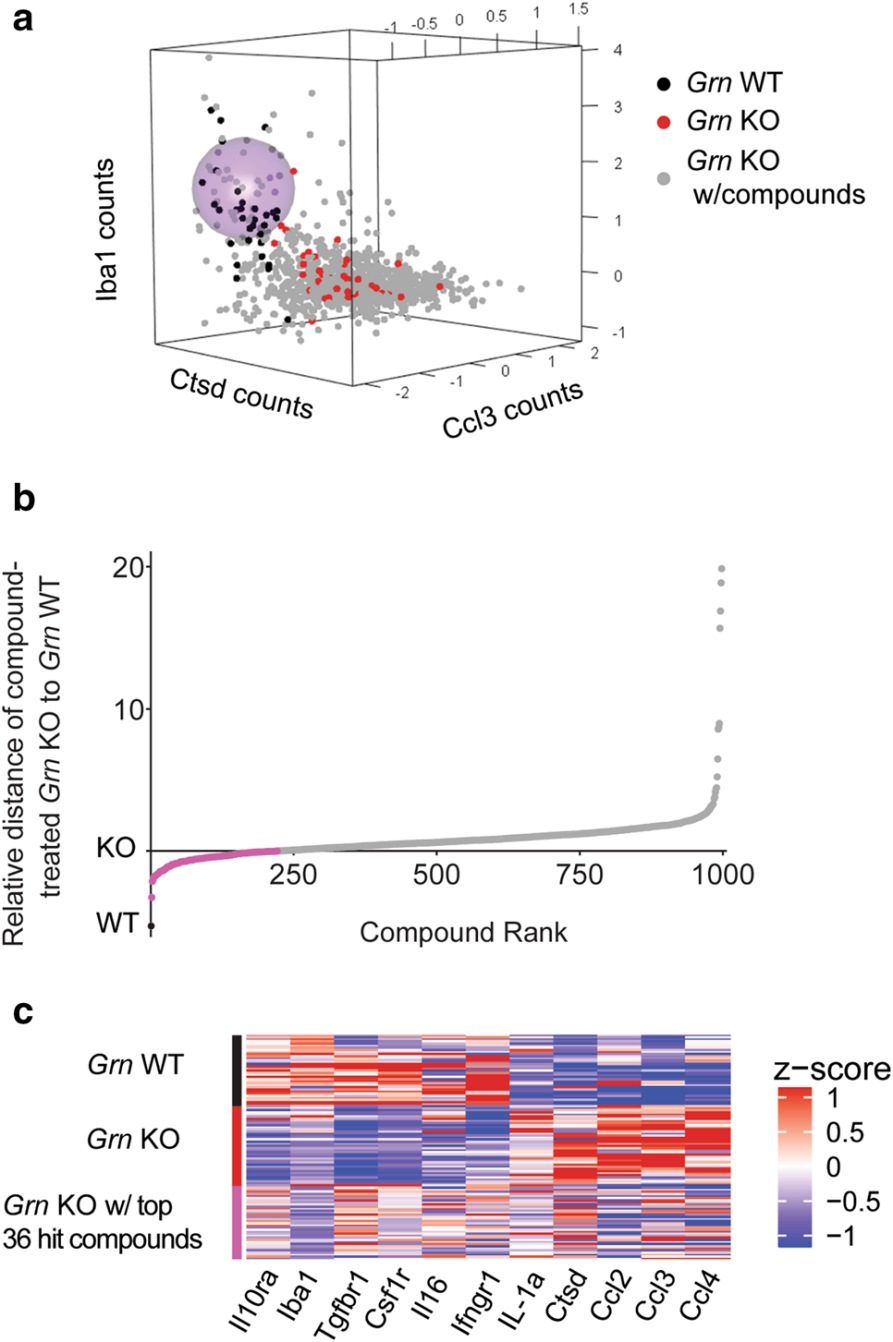
Identification of compounds that shift transcriptional profile of *Grn* KO to *Grn* WT microglia cells. (**a**) 3D plot representing normalized transcriptional profiles of all conditions for three genes to demonstrate distance of radius 0.75 (light purple sphere) around the center of the WT cells. (**b**) Euclidian distance with compounds ranked from closest to farthest away from the WT, with KO at 0; conditions below 0 are shaded in pink. (**c**) Heatmap representing *Grn* WT, KO, and the top compound-treated 36 wells.

The distance metric can be illustrated in three dimensions, with normalized (mean at 0, sd = 1) counts graphed for *Ctsd* on the x-axis, *Ccl3* on the y-axis, and *Iba1* on the z-axis (Fig. 2a). As an example, we used a radius of 0.75 distance units (in normalized counts) from the *Grn* WT center to illustrate compounds that shift the *Grn* KO cells toward the *Grn* WT transcriptional profile in the three chosen dimensions. This area is encompassed by a light purple sphere (Fig. 2a). Similarly, by calculating the distance in 11 dimensions (where each gene is a dimension), the compounds can be ordered (x-axis) based on their distances away from the *Grn* WT center (y-axis), from closest to farthest away, with KO arbitrarily set to 0. The distance of the compound-treated KO cells to the WT cells ranged from 2.01 to 25.15, with mean of 6.02 (Fig. 2b). Compounds whose distances from the WT average are less than the distance between WT and KO cells are represented in pink in Fig. 2b. We considered these 220 compounds as hit compounds for further evaluation. The top 36 hit compounds altered the transcriptional profiles of *Grn* KO cells such that they were much closer to those of the *Grn* WT cells (Fig. 2c). In the absence of progranulin expression, the normalization of the profile of selective signature genes indicates that these compounds act as *progranulin transcriptional mimetics*.

### Transcriptional corrector compounds partially rescue cysteine cathepsin dysfunction in progranulin-deficient primary mouse microglia

**Figure 3:**
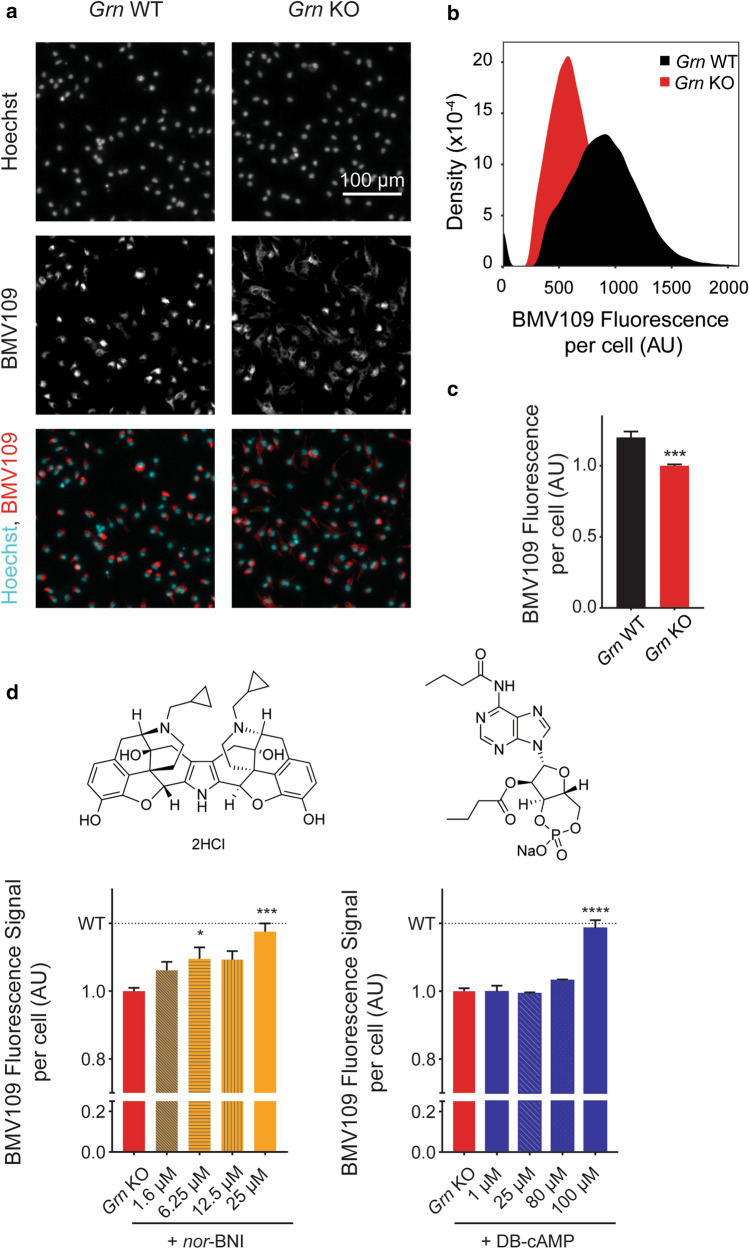
Cysteine cathepsin activity is partially rescued with addition of transcriptional correctors to *Grn* KO microglia. (**a**) Fluorescent images of BMV109 and Hoechst in *Grn* WT and KO cells (Scale bar at 100 μm). (**b**) Distribution of signal per cell for *Grn* WT and KO microglia from (**a**). (**c**) Quantification of 4 independent experiments, (mean ± SEM), WT n = 21 wells and KO n = 21 wells, unpaired t-test with Welch’s correction in GraphPad Prism v8.11, ***p value ≤ 0.001. (**d**) BMV109 signal quantification with increasing concentration of *nor*-BNI and DB-cAMP, (mean ± SEM); quantification for nor-BNI: 3 independent experiments, n = 3–14 wells for each condition, one-way Kruskal Wallis non parametric ANOVA with Dunn’s multiple comparisons comparing each condition to *Grn* KO in GraphPad Prism v8.11, *p value ≤ 0.05, ***p value ≤ 0.001; DB-cAMP: 4 independent experiments, n = 3–20 wells for each condition, one-way ANOVA with Dunnett’s multiple comparisons comparing each condition to *Grn* KO in GraphPad Prism v8.11, ****p value ≤ 0.0001.

To investigate the ability of the hit compounds to change the phenotype of microglial cells, as well as to further narrow down compound candidates, we determined their effect on lysosomal activity. We previously reported that human derived fibroblasts from patients with progranulin haploinsuffiency have impaired lysosomal function compared to cells from their siblings^5^. Taking advantage of the fluorescence-based cathepsin probe BMV109^28^, which measures cathepsin activity directly, we established an assay to determine how the top hits from the RASL screen affect cysteine cathepsin activity in progranulin-deficient microglia. Reduced fluorescence of the BMV109 probe indicates reduced cathepsin activity. The specificity of this assay was demonstrated by the weak cathepsin B and L inhibitor Z-Phe-Ala-diazomethylketone (PADK)^29,30^, which had a concentration-dependent lowering effect on BMV109 fluorescence intensity (Fig. S2). We performed live imaging on *Grn* WT and *Grn* KO cells labeled with BMV109 and Hoechst as a nuclear counter stain (Fig. 3a). *Grn* KO cells had much lower BMV109 signal than WT (Fig. 3b,c), in agreement with our findings in patient-derived fibroblasts^5^. Notably, this impairment in lysosomal function occurred despite the increased mRNA level of the lysosomal protease cathepsin D in *Grn* KO microglia (Fig. 1b), consistent with previous findings in *Grn* KO mice^21^ and neurons derived from FTD patients^31^.

Next, we tested 55 of the lead compounds from the RASL-seq screen in the BMV109 cysteine cathepsin activity assay. The two compounds with the strongest rescue of the BMV109 fluorescence intensity were *nor*-binaltorphimine dihydrochloride (*nor*-BNI) and dibutyryl-cAMP, sodium salt (DB-cAMP). Indeed, at the highest concentrations tested, both *nor*-BNI and DB-cAMP raised the BMV109 fluorescence to WT levels (Fig. 3d) without exhibiting cytotoxicity (Fig. S3). These two compounds were chosen for further analysis to investigate their full effect on mRNA levels of *Grn* KO cells in mouse microglia.

### Compounds rescue cell cycle-related transcriptional alternations induced by progranulin deficiency

**Figure 4:**
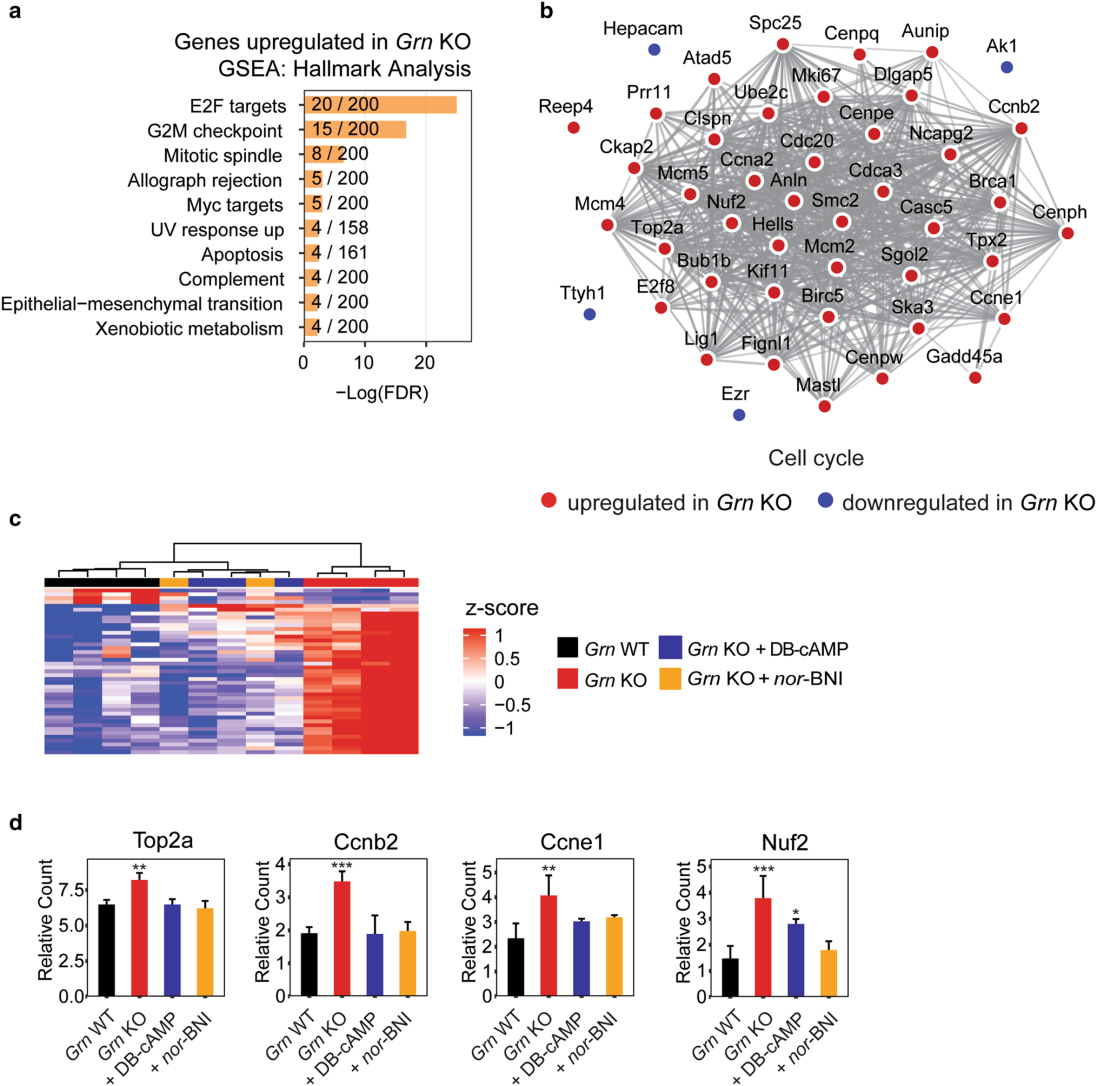
Compounds rescue cell cycle dysregulation in *Grn* KO mouse microglia. (**a**) Hallmark GSEA analysis of upregulated genes in *Grn* KO microglia. (**b**) Cytoscape network of cell cycle genes dysregulated in *Grn* KO microglia. (**c**) Heatmap and unbiased column clustering (Ward’s method with Euclidean distance) of cell cycle genes from (**b**) demonstrates that compound addition to *Grn* KO cells normalized expression toward *Grn* WT cells. (**d**) mRNA expression of individual genes shows compound addition to *Grn* KO cells normalized expression toward the *Grn* WT cell levels. n = 2–4; *p value ≤ 0.05, **p value ≤ 0.01, ***p value ≤ 0.001.

To gain a more comprehensive understanding of the transcriptional dysregulation caused by *Grn* deficiency, we performed RNA-seq on *Grn* KO and WT primary microglia. We identified 113 upregulated and 83 downregulated genes in *Grn* KO microglia (FC ≥ 2 or FC ≤ -2, *p* value ≤ 0.005). Among the upregulated genes in *Grn* KO microglia, we observed a striking enrichment in cell cycle-related genes, with 6 of the top 10 categories connected to the cell cycle: E2F targets, G2M checkpoint, mitotic spindle, Myc targets, apoptosis, and epithelial − mesenchymal transition (Fig. 4a). Visualization of all differentially expressed genes using Cytoscape’s STRING database further validated the overrepresentation of cell cycle genes, highlighting 45 genes in the cell cycle process that have known interactions (Fig. 4b). While this striking transcriptional alteration of cell cycle genes has not been previously reported in progranulin-deficient cells, these findings are consistent with the long-standing observation that progranulin-deficient microglia exhibit microgliosis, characterized by increased proliferation, in the adult mouse brain^32^. Using BrdU to identify proliferating cells, we confirmed that *Grn*^*–/–*^ cells exhibited significantly increased proliferation (Fig. S4).

We then performed RNA-seq analyses of progranulin deficient microglia treated with *nor*-BNI or DB-cAMP. Unbiased hierarchical analysis of *nor*-BNI and DB-cAMP-treated samples revealed that treatment with these compounds markedly shifted the expression of cell cycle genes, such that the compound-treated cells clustered with *Grn* WT cells (Fig. 4c). Examining individual cell cycle genes, we observed that the expression levels of *Top2a*, *Ccnb2*, *Ccne1,* and *Nuf2* were significantly increased in KO microglia, and that treatment with either *nor*-BNI or DB-cAMP reduced transcript expression to a level equivalent to WT (Fig. 4d).

Among the 83 downregulated genes, the inflammatory response was the most dysregulated pathway (FDR 3.88 × 10^−5^) (Fig. S5). Visually representing STRING-defined immune system process genes on Cytoscape (Fig. S5) revealed a mix of up and downregulated genes with several known connections. For example, there was dowregulation of *Cx3cr1* and *Il1b*, and upregulation in *Cd28*, *Cd38*, and *Cd80* in the *Grn* KO. We next examined the effect of *nor*-BNI and DB-cAMP on the expression of these genes by performing unbiased hierarchical clustering. Overall, *nor*-BNI and DB-cAMP had a modest effect on gene expression, as illustrated by the heatmap showing *Grn* WT clustering separately from the other conditions (Fig. S5). Upon inspection of individual genes, we observed modest normalization of some genes (e.g. *Anln* and *Rsad2*), while transcription levels of many others were not changed in response to these compounds (e.g., *Ccl9* and *Cx3cr1*) (Fig. S5).

The RNA-seq analyses also identified a dysregulation in several of lysosomal genes that have been previously examined in the *Grn* KO model^33^. Compared to WT cells, the *Grn* KO cells had increased expression of *Atp6v0d2* and *Atp6ap*, which are involved in lysosomal acidification, and increased expression of *Cd63*, a lysosomal membrane gene. On the other hand, expression of the lysosomal hydrolase gene *Hexb*, a microglia-enriched gene that is part of the microglial sensome^27^, was decreased (Fig. S6). Treatment of *Grn* KO cells with *nor*-BNI normalized *Hexb* gene expression, but the expression of other lysosomal genes analyzed was not altered by *nor*-BNI nor by DB-cAMP (Fig. S6). Comparing the effects of these two compounds on *Grn* KO cells, we found a substantial (over 50%) overlap of downregulated genes and shared pathways, including G2M checkpoint, E2F Targets, mitotic spindle, and Tnf-α signaling via NF-κB (Fig. S7), suggesting converging mechanisms. However, differential mechanisms are likely to be involved with limited overlap in upregulated genes and commonality in the pathways affected (Fig. S7).

## Discussion

Our study demonstrates how a dual-platform approach utilizing RASL-seq and phenotypic screening leads to discovery of compounds that are both transcriptional and functional correctors in a cellular model of dementia. This approach is particularly applicable to target microglia because they exhibit different profiles due to environmental and genetic variability in health and disease. We identified compounds that not only rescue cysteine cathepsin dysfunction, but also shift the transcriptional profile of *Grn* KO microglia closer to that of *Grn* WT microglia. Using RASL-seq, we screened a library of bioactive compounds to find small molecules that normalized inflammatory, microglial, and lysosome gene expression of *Grn* KO cells. We identified 220 hits that shifted the expression profile of a subset of gene signatures of *Grn* KO microglia to that of WT microglia. We then utilized a second screen to further narrow down hit compounds that improved lysosomal function of microglia. We discovered that two compounds, *nor*-BNI and DB-cAMP, increased cysteine cathepsin function of *Grn* KO cells to the level of the WT cells. We then assessed the effects of compounds on the whole transcriptomic level, and identified the cell cycle network as a major, novel dysregulated pathway in *Grn* KO microglia that was largely normalized by treatment with *nor*-BNI and DB-cAMP.

Microglia have recently emerged as targets for neurodegenerative diseases, with several new clinical efforts underway^34^. At the same time, there is a growing appreciation that the microglial transcriptome and proteome are complex and pathology-, age-, and location-dependent. We demonstrated that the RASL-seq approach is particularly suitable to screening compounds based on a multi-gene signature, and apply this method in *Grn* KO microglia. The transcripts we screened for contained multiple lysosomal genes, some of which were found to be markedly elevated in progranulin knockout brains^14^. Lysosomal enzymatic activity was also found to be significantly diminished in progranulin haploinsufficient human fibroblasts^5^ and progranulin deficient mouse microglia (Fig. 3a–c). However, we showed that the lysosomal gene network was surprisingly largely unaffected in *Grn* KO microglia, suggesting that post-transcriptional regulation, such as protein clearance and trafficking, may underlie the aberrations in lysosomal function observed in *Grn* KO microglia. Progranulin is localized in the lysosome and secreted extracellularly, which could exhibit different biological functions.

Our RNA-seq results indicate that there is a clear dysregulation of the cell cycle in *Grn* KO mouse microglia. Interestingly, cell cycle dysregulation was recently identified in single cell RNA-seq analysis of microglia from a mouse model of Alzheimer’s disease (AD), with some of the same genes identified in our study, including *Top2a*, *Hells*, *Ccne1*, *Spc25*, *Cenpe*, *Anln*, *Rsad2*, and *Ifitm3*^35^. Neuronal cell cycle re-entry is a known feature of AD^36^, and cell cycle dysregulation has been reported in FTD-GRN patient lymphoblasts^37^. On the other hand, overabundance of progranulin has a proliferative effect, as it is considered to be a growth factor that contributes to tumorigenesis^38^. The mechanism underlying this dichotomy is unclear. It is possible that regulation of the cell cycle by progranulin could depend on cellular environments, and differ between progranulin and its cleaved products, granulins. Nevertheless, our data suggest that an aberrant cell cycle in microglia may also play an important role in progranulin-deficient FTD.

Our combined RASL-seq and cysteine cathepsin activity assay yielded two compounds that not only enhanced lysosomal function, but also largely normalized the dysregulated cell cycle genes in progranulin-deficient microglia. The first compound, *nor*-BNI is a κ-opioid receptor antagonist^39,40^ that was previously investigated in stress and depression models in rodents^41–43^, as well as drug addiction models in rodent and non-human primates^44,45^. Thus, our findings that *nor*-BNI regulates the cell cycle and lysosomal activity in microglia are surprising, and further studies are warranted to elucidate the molecular mechanism underlying those effects. In contrast, DB-cAMP, which is a cell-permeable analog of cAMP^46^, has been shown to have anticancer properties; it reduced tumorigenesis in animal models and halted proliferation in cells lines^47^, including rat glioma cells^48^. The anti-proliferative effect of DB-cAMP has been difficult to replicate^47^, with confounding contribution of the metabolite butyrate^46^. DB-cAMP has also been shown to reduce TNF-α and increase IL-1β and IL-10 protein and mRNA levels in lipoposaccharide-treated BV2 cells^49^, and to reduce CCL2 production in macrophages activated by INF-γ and the TLR2 ligand glycosylphosphatidylinositol-anchored mucinlike glycoproteins (tGPI-mucins)^50^. Consistent with these data, we observed that DB-cAMP regulated the cell cycle, and RNA-seq revealed an increase in *Il-1β* transcript level, while *Tnf-*a, *IP10*, and *Ccl2* levels were not significantly altered in the compound-treated *Grn* KO cells compared to the untreated KO controls.

Our study uncovered a potential link between lysosomal dysfunction and the cell cycle in neurodegeneration. The lysosome is part of the autophagy process. In cancer, autophagy can play a dual role; while dysregulation of the autophagic process is associated with increased incidence of cancer, autophagy is also required for sustained growth of tumors^51,52^. Since progranulin has a known role in the lysosome^53,54^, the link between lysosomal function and cell cycle dysregulation in progranulin deficient microglia warrants further investigation.

In summary, we established a multi-tiered drug discovery platform that harnesses the multiplex transcriptomic ability of RASL-seq, combined with functional assays and pharmacogenomics. Our unique drug discovery platform is well positioned to identify novel microglial modulators in neurodegenerative diseases.

## Methods

### Mice

WT control C57BL/6 mice (obtained from the National Institute on Aging) and complete *Grn* KO mice^55^ were used in these experiments. All mice were housed with ad libitum access to food and water in a pathogen-free barrier facility with a 12-h light/dark cycle. Animal experimental protocols were approved by the Institutional Animal Care and Use Committee of the University of California, San Francisco, and all methods were carried out in accordance with relevant guidelines and regulations.

### Generation of primary mouse microglia

Cortical culture was prepared from isolated cortex tissue of postnatal day 3 *Grn* WT and KO pups and grown in T75 PDL-coated flasks in DMEM supplemented with 10% fetal bovine serum (FBS), 100 units/mL penicillin, and 100 μg/mL streptomycin at 37° C with 5% CO_2_. After 10 days, the flasks were shaken for 2 h at 200 rpm at 37° C, the media were collected in 25 mL Falcon tubes, which were spun at 200 g for 15 min. Media were removed and microglia were resuspended in 1 mL (per 75 T flask) and counted using a hemocytometer. Microglia were plated for three separate experiments: at 7,000 cells/well in 50 μL into 384-well plates (Corning 3,707) for RASL-seq experiment, at 30,000 cells/well in 100 μL into 96-well plates (TPP Sigma Z707910) for BMV109 experiment, and at 300,000 cells/well in 1 mL into 24-well plates for RNA-seq experiments.

### RASL-seq

RNA-mediated oligonucleotide Annealing, Selection, and Ligation with Next-Gen sequencing (RASL-seq) platform was used as described in the literature^16^, with the following modifications. Compound library used was #2,884 Tocriscreen Total, which includes 1,120 biologically active compounds dissolved in DMSO at 10 mM concentration, distributed among four 384-well plates. For compound treatment, 50 nL of compound-containing DMSO was added to microglia-containing wells, and plates were incubated for 24 h. Medium was removed from each well and the cells were washed twice with 1.6 μM Hoechst-containing non-fluorescent DMEM. The plates were imaged using a fluorescence plate reader to determine cell count. Subsequently, the cells were lysed prior to the annealing, selection, and ligation steps of RASL-seq. Three probe sets of 69 gene targets were designed and tested in pilot studies. Out of these, 42 mRNA probes, including 6 housekeeping genes, were used for the RASL-seq compound screen (Table S1). After barcoding and amplification, the probe pools for all wells were combined and sequenced on the HiSeq 4,000 System at UCSF’s Center for Advanced Technology.

### Ranking of RASL-seq compound hits

Treated and non-treated wells with cell count below 40% of average for controls were removed from further analysis. As well, samples with low total reads (< 100,000) and probes with low total reads for each plate (< 2,000) were removed. Counts from each plate were then normalized to the sum of housekeeping genes. For analysis of compound potency, data for each of the four plates were combined. Each plate contained non-treated wells of each genotype. To define the signature gene profile, genes with expression difference of > 1.5 fold with *p* value < 0.05 were considered. One *Grn* WT well did not unbiasedly cluster (Ward’s method with Euclidian distance) with its group, and was taken out of the heatmaps in Figs. 1c and 2c, as well as the analysis of compounds effect on *Grn* KO cells.

For compound ranking, R^56^ (complete list and references for packages are included in Supplementary Methods) was used to calculate Euclidean distance in 11 dimensions (corresponding to each gene in the profile) from each compound-treated well to the mean of the *Grn* WT cells, according to Eq. (1).

1 $$Euclidean \; distance = \sqrt {\mathop \sum \limits_{i = 1}^{11} \left( {q_{i} - p_{i} } \right)^{2} }$$

### Compounds

Compounds used in activity assay were repurchased: nor-binaltorphimine dihydrochloride (Tocris 0347) and dibutyryl-cAMP, sodium salt (Tocris 1141). PADK was purchased from Bachem Americas, Inc., Torrance, CA (N-1040). BMV109 was obtained from Matt Bogyo and from Vergent Bio as IABP Pan Cathepsin Activity Based Probe.

### Cathepsin activity assay with BMV109

The cathepsin activity assay was performed as previously described^5^ with some modifications. The plated microglia in 96-well TPP plates were incubated for 24 h in growth medium, the media were replaced with 100 μL media including dissolved compound or vehicle (final concentration of DMSO was 0.1%). After 24 h of incubation, the media were aspirated and 75 μL of 0.25 μM BMV in media were added. The probe was incubated for 1 h and the media were removed. The cells were washed with non-fluorescent DMEM that contained 1.6 μM Hoechst stain three times. Imaging was performed with Cellomics ArrayScan XTI (Thermo Fisher) AquireOnly.V3 protocol at 10 × magnification with 2 × 2 binning using two channels: 386 nM for Hoechst, 0.02 s exposure (Channel 1) and at 650 nm for BMV109, 0.1 s exposure (Channel 2). The acquired images were analyzed using HCS Studio Cell Analysis Software, HealthCellProfiling protocol, with details in Supplementary Methods. Comparisons of cell count and BMV109 signal was performed using GraphPad Prism (version 8.11 for Windows, GraphPad Software, La Jolla California USA, www.graphpad.com) on mean values per well; experiments performed on different days were combined by normalizing all data to the *Grn* KO mean, which was set at 1. Representative images for each of the two channels were exported from Cellomics Software iView as 16 bit tiffs; ImageJ^57^ was used to add the scale bar, and the images were pseudo colored and uniformly thresholded for illustrative purposes.

### RNA sequencing

The plated cells were incubated for 24 h in media prior to media replacement with either growth media containing compounds (*nor*-BNI at 25 μM, DB-cAMP at 100 μM, final DMSO concentration 0.1%) or 0.1% DMSO vehicle control. After another 24 h, the media was aspirated and the cells were washed with cold PBS three times. The mRNA from cells was isolated using the Quick-RNA MiniPrep Kit from Zymo Research (catalog No. R1055), and further purified and concentrated using RNA Clean&Concentrator-25 kit from Zymo Research (catalog No. R1017). To measure RNA concentration and quality, NanoDrop and Agilent RNA Pico Chip on Bioanalyzer were used, respectively. Samples with RIN # above 7 were considered to be good quality and their libraries were prepared in two batches following the protocol from Lexogen QuantSeq 3′mRNA-Seq Library Prep Kit FWD for Illumina. The library quality was assessed using Agilent High Sensitive DNA Chip on Bioanalyzer and the library concentrations were measured using Qubit dsDNA HS Assay Kit. The individual libraries were pulled together and submitted for sequencing on the HiSeq 4,000 at SE50 at the Center for Advanced Technology at the University of California, San Francisco.

### RNA-sequencing data analysis

The RNA-seq read mapping and read count was performed using the Lexogen QuantSeq Platform on the Bluebee cloud (https://www.bluebee.com/lexogen/) with GRCm38.84 reference genome. Samples had an average of 65% mapped reads (55% for one batch and 70% for second batch); one sample (*Grn* KO + *nor*-BNI) had a mapped read rate below others in the batch (33%) and was removed from analysis. Low expressing genes were filtered out, leaving genes with more than 10 counts in at least 3 conditions for further analysis. The counts from the two batches were normalized using RUVSEq package in R^58^ with the upper-quartile normalization followed by replicate sample normalization with two factors of unwanted variation (k = 2). Samples that were in common between the two batches were used as controls to ensure batch normalization. Sample comparison was performed using the DESeq2^59^ package in R using 4 groups (*Grn* WT, *Grn* KO, *Grn* KO + *nor*-BNI, *Grn* KO + DB-cAMP). Differentially expressed (DE) genes were those that had a log2FC difference of ≥ 1 or ≤ -1 and *p* value ≤ 0.005. The subsequent network analyses were performed using the online tools GSEA v6.3^60,61^ and STRING v11.0^62^, as well as Cytoscape software v3.6.1^63^. For GSEA, upregulated and downregulated gene lists were used for input to analyze using GSEA: Hallmark analysis^64^; combined genes lists were used on STRING to classify according to GO Biological Process Gene Ontology (GO)^65,66^ using default settings and these groups were input into Cytoscape for generating figures with the default setting of confidence for edges at 0.4 and expression data for node color coding.

### BrdU Cell Proliferation

WT (WTC11) and isogenic *GRN*-null (7 bp ins/10 bp del) induced pluripotent stem cells (iPSCs) were cultured according to a previously published method^67^. BrdU cell proliferation assay was performed using BrdU Cell Proliferation ELISA Kit (colorimetric) from Abcam (catalog No. ab126556) on iPSCs at 3 and 4 days after plating at 40,000 cells/well (n = 3) in 96-well plate (40 and 80% confluence; passages 40 and 43; respectively).

#### Statistical analysis

Statistical analyses were performed with GraphPad Prism (version 8.11 for Windows, GraphPad Software, La Jolla California USA, www.graphpad.com) and R packages (complete list in Supplementary Methods). In Fig. 3c, BMV109 signal per-well data set passed Shapiro–Wilk normality test and unpaired t-test with Welch’s correction was performed in GraphPad; n = 21 wells from 4 independent experiments for both WT and KO conditions. In Fig. 3d, BMV109 signal per-well data sets for *Grn* KO and *nor*-BNI did not pass Shapiro–Wilk normality test and Kruskal Wallis non parametric ANOVA with Dunn’s multiple comparisons was performed in GraphPad, n = 14 for *Grn* KO, n = 3–5 for each concentration of *nor*-BNI; BMV109 signal per-well data set for DB-cAMP passed Shapiro–Wilk normality test and ANOVA with Dunnett’s multiple comparisons was performed in GraphPad, n = 20 for *Grn* KO, n = 3–8 for each concentration of DB-cAMP. In Fig. S2, BMV109 signal per-well data set for PADK passed normality test and ANOVA with Dunnett’s multiple comparisons was performed in GraphPad. In Fig. S3, toxicity data passed normality test and ANOVA with Dunnett’s multiple comparisons was performed in GraphPad comparing the untreated *Grn* KO control to each compound-treated *Grn* KO condition. In Figs. 4, S5, and S6, *p* values for each gene were obtained from the multiple comparison analysis using the DESeq2 package in R.

## Supplementary information

Supplementary information 1.

Supplementary information 2.

Supplementary information 3.

Supplementary information 4.

## Data Availability

The RASL-seq dataset is available as Supplementary Data. The mRNA-seq dataset is available at Gene Expression Omnibus, GSE143144.
