# Supplementary material for: Discovery of small molecules that normalize the transcriptome and enhance cysteine cathepsin activity in progranulin-deficient microglia
Source: Sci Rep. 2020 Aug 13;10:13688. doi: 10.1038/s41598-020-70534-9 (PMC7426857; doi:10.1038/s41598-020-70534-9)
Supplement: Supplementary file 4 — Supplementary information 4. [file 41598_2020_70534_MOESM4_ESM.pdf]

Supporting materials for:

Discovery of small molecules that normalize the transcriptome and enhance cysteine cathepsin activity in progranulin-deficient microglia

Maria A. Telpoukhovskaia[1,2], Kai Liu[3], Faten A. Sayed[1,2,4], Jon Iker Etchegaray[1], Min Xie[1,3], Lihong Zhan[1,2], Yaqiao Li[1], Yungui Zhou[1], David Le[1], Ben A. Bahr[5], Matthew Bogyo[6], Sheng Ding[3], Li Gan[1,2,7,8,*]

[1]Gladstone Institute of Neurological Disease, San Francisco, CA, 94158, USA

[2]Department of Neurology, University of California, San Francisco, CA, 94158, USA

[3]Gladstone Institute of Cardiovascular Disease, San Francisco, CA, 94158, USA

[4]Neuroscience Graduate Program, University of California, San Francisco, CA, 94158, USA

[5]Biotechnology Research and Training Center, University of North Carolina at Pembroke, Pembroke, NC, 28372, USA

[6]Department of Pathology, Stanford University, CA, 94305, USA

[7]Helen and Robert Appel Alzheimer's Disease Institute, Brain and Mind Research Institute, Weill Cornell Medicine, New York, NY, 10021, USA

[8]Lead Contact

*Correspondence: lig2033@med.cornell.edu.

Supplementary Table S1. Targeted genes, probe sequences, and probe concentrations used for the RASL-seq experiment. * denotes a housekeeping gene

Gene name	mRNA targeted sequence, probe sequences	Concentration (nM)
adar_3*	**ACCAGAGGCACTGTGGATGGACCAGGGAAAGAGTTGTCTC**<br>ACACGTTCAGAGTTCTACAGTCCGACGATCGAGACAACTCTTTCCCTGGT<br>/5Phos/CCATCCACAGTGCCTCTGGTTGGAATTCTCGGGTGCCAAGGAACTCCAGTCAC	150
agpat1_2*	**ACCTTGCAGTACAGGCCCAGGTCCCCATCATCCCCATCGT**<br>ACACGTTCAGAGTTCTACAGTCCGACGATCACGATGGGGATGATGGGGAC<br>/5Phos/CTGGGCCTGTACTGCAAGGTTGGAATTCTCGGGTGCCAAGGAACTCCAGTCAC	100
atp5o_3*	**TACTGAAACTGGAGATCAAGACTGACCCGTCAATCATGGG**<br>ACACGTTCAGAGTTCTACAGTCCGACGATCCCCATGATTGACGGGTCAGT<br>/5Phos/CTTGATCTCCAGTTTCAGTATGGAATTCTCGGGTGCCAAGGAACTCCAGTCAC	50
Bcat2_3*	**AAATCCTGTATGAAGGCAAGCAACTCCACATACCTACCAT**<br>ACACGTTCAGAGTTCTACAGTCCGACGATCATGGTAGGTATGTGGAGTTG<br>/5Phos/CTTGCCTTCATACAGGATTTTGGAATTCTCGGGTGCCAAGGAACTCCAGTCAC	200
C3ar1_2	**TTCTGGGCATAAGACTGCAGTTCTGCCTTTCCAGTATGAG**<br>/5Phos/CTGCAGTCTTATGCCCAGAATGGAATTCTCGGGTGCCAAGGAACTCCAGTCAC<br>ACACGTTCAGAGTTCTACAGTCCGACGATCCTCATACTGGAAAGGCAGAA	200
Ccl2_3	**AGGTGTGGATCCATTTTTCCCTCTCTGTGAATCCAGATTC**<br>ACACGTTCAGAGTTCTACAGTCCGACGATCGAATCTGGATTCACAGAGAG<br>/5Phos/GGAAAAATGGATCCACACCTTGGAATTCTCGGGTGCCAAGGAACTCCAGTCAC	50
Ccl3_3	**TGCCAAGTAGCCACATCGAGGGACTCTTCACTTGAAATTT**<br>ACACGTTCAGAGTTCTACAGTCCGACGATCAAATTTCAAGTGAAGAGTCC<br>/5Phos/CTCGATGTGGCTACTTGGCATGGAATTCTCGGGTGCCAAGGAACTCCAGTCAC	20
Ccl4_1	**TGCCTCTTTTGGTCAGGAATACCACAGCTGGCTTGGAGCA**<br>ACACGTTCAGAGTTCTACAGTCCGACGATCACGGAGAAAACCAGTCCTTA<br>/5Phos/TGGTGTCGACCGAACCTCGTTGGAATTCTCGGGTGCCAAGGAACTCCAGTCAC	200
Cd11b_2	**GGCTCCACTTTGGTCTCTGTCTTAGACCTCACATACGACT**<br>ACACGTTCAGAGTTCTACAGTCCGACGATCCCGAGGTGAAACCAGAGACA<br>/5Phos/GAATCTGGAGTGTATGCTGATGGAATTCTCGGGTGCCAAGGAACTCCAGTCAC	20
Csf1r_2	**GTATTTGCACCGAAGAACATATACAGCATCATGCAGTCCT**<br>/5Phos/ATGTTCTTCGGTGCAAATACTGGAATTCTCGGGTGCCAAGGAACTCCAGTCAC<br>ACACGTTCAGAGTTCTACAGTCCGACGATCAGGACTGCATGATGCTGTAT	30
Ctsd_3	**GATACTTGACATAGCCTGCTGGGTCCACCATAAGTACAAC**<br>/5Phos/AGCAGGCTATGTCAAGTATCTGGAATTCTCGGGTGCCAAGGAACTCCAGTCAC<br>ACACGTTCAGAGTTCTACAGTCCGACGATCGTTGTACTTATGGTGGACCC	50
Cxcl1_1	**TCCGTTACTTGGGGACACCTTTTAGCATCTTTTGGACAAT**<br>ACACGTTCAGAGTTCTACAGTCCGACGATCAGGCAATGAACCCCTGTGGA<br>/5Phos/AAATCGTAGAAAACCTGTTATGGAATTCTCGGGTGCCAAGGAACTCCAGTCAC	250
Dap12_2	**AGGTCAAGGGACAGCGGAAGGGACCCGGAAACAACACATT**<br>/5Phos/CTTCCGCTGTCCCTTGACCTTGGAATTCTCGGGTGCCAAGGAACTCCAGTCAC	50

	ACACGTTCAGAGTTCTACAGTCCGACGATCAATGTGTTGTTTCCGGGTCC	
Eea1_3	**AGAAGAGCACTCCTAGAGAGGTGTCTTAAAGGCGAAGGTG**<br>/5Phos/CTCTCTAGGAGTGCTCTTCTTGGAATTCTCGGGTGCCAAGGAACTCCAGTCAC<br>ACACGTTCAGAGTTCTACAGTCCGACGATCCACCTTCGCCTTTAAGACAC	100
Grn_1	**CCTGCTGTCCCTACCTAAAGGGTGTCTGCTGTAGAGATGG**<br>/5Phos/CTTTAGGTAGGGACAGCAGGTGGAATTCTCGGGTGCCAAGGAACTCCAGTCAC<br>ACACGTTCAGAGTTCTACAGTCCGACGATCCCATCTCTACAGCAGACACC	50
Hspa8_2	**CACCATCACCAATGACAAGGGCCGCTTGAGTAAGGAAGAT**<br>/5Phos/CCTTGTCATTGGTGATGGTGTGGAATTCTCGGGTGCCAAGGAACTCCAGTCAC<br>ACACGTTCAGAGTTCTACAGTCCGACGATCATCTTCCTTACTCAAGCGGC	30
Iba1_1	**TCTCCTCATACATCAGAATCATTCTCAAGATGGCAGATCT**<br>ACACGTTCAGAGTTCTACAGTCCGACGATCAGAGGAGTATGTAGTCTTAG<br>/5Phos/TAAGAGTTCTACCGTCTAGATGGAATTCTCGGGTGCCAAGGAACTCCAGTCAC	50
Ifngr1_3	**AGCATAACCGGAGTGGGGAGATCCTACATACGAAACATAC**<br>/5Phos/CAAGGACTTAGGTAACATTATGGAATTCTCGGGTGCCAAGGAACTCCAGTCAC<br>ACACGTTCAGAGTTCTACAGTCCGACGATCGCACTTTTTACCACAGAGAG	100
Il10ra_3	**GGAAGTTCTCAGAACTAAAGAATGCAACCAAGAGAGTGAA**<br>/5Phos/CTTTAGTTCTGAGAACTTCCTGGAATTCTCGGGTGCCAAGGAACTCCAGTCAC<br>ACACGTTCAGAGTTCTACAGTCCGACGATCTTCACTCTCTTGGTTGCATT	200
Il15_2	**ACACTGACAGTGACTTTCATCCCAGTTGCAAAGTTACTGC**<br>ACACGTTCAGAGTTCTACAGTCCGACGATCGCAGTAACTTTGCAACTGGG<br>/5Phos/ATGAAAGTCACTGTCAGTGTTGGAATTCTCGGGTGCCAAGGAACTCCAGTCAC	300
Il16_1	**CATAAACAGGATTTTCAAAGGGACAGAACAGGGTGAGATG**<br>/5Phos/CTTTGAAAATCCTGTTTATGTGGAATTCTCGGGTGCCAAGGAACTCCAGTCAC<br>ACACGTTCAGAGTTCTACAGTCCGACGATCCATCTCACCCTGTTCTGTCC	300
Il-1α_1	**TTTGGTGTTTCTGGCAACTCCTTCAGCAACACGGGCTGGT**<br>ACACGTTCAGAGTTCTACAGTCCGACGATCAAACCACAAAGACCGTTGAG<br>/5Phos/GAAGTCGTTGTGCCCGACCATGGAATTCTCGGGTGCCAAGGAACTCCAGTCAC	300
Il-1β_3	**CACCCACCCTGCAGCTGGAGAGTGTGGATCCCAAGCAATA**<br>ACACGTTCAGAGTTCTACAGTCCGACGATCTATTGCTTGGGATCCACACT<br>/5Phos/CTCCAGCTGCAGGGTGGGTGTGGAATTCTCGGGTGCCAAGGAACTCCAGTCAC	250
mIP10_1	**GTTAAGGAGCCCTTTTAGACCTTTTTTGGCTAAACGCTTT**<br>ACACGTTCAGAGTTCTACAGTCCGACGATCCAATTCCTCGGGAAAATCTG<br>/5Phos/GAAAAAACCGATTTGCGAAATGGAATTCTCGGGTGCCAAGGAACTCCAGTCAC	300
Lamp1_3	**TGGAATTGCAGTTTGGGATGAATGCCAGCTCTAGCCTGTT**<br>/5Phos/CATCCCAAACTGCAATTCCATGGAATTCTCGGGTGCCAAGGAACTCCAGTCAC<br>ACACGTTCAGAGTTCTACAGTCCGACGATCAACAGGCTAGAGCTGGCATT	100
Lamp2_2	**GTATTTGGCTAATGGCTCAGCTTTCAACATTTCCAACAAG**<br>/5Phos/CTGAGCCATTAGCCAAATACTGGAATTCTCGGGTGCCAAGGAACTCCAGTCAC<br>ACACGTTCAGAGTTCTACAGTCCGACGATCCTTGTTGGAAATGTTGAAAG	100
Lrp1_1	**TTGCCCTTGACCCTGACAAGCCTACCAACTTCACCAACCC**	100

	/5Phos/CTTGTCAGGGTCAAGGGCAATGGAATTCTCGGGTGCCAAGGAACTCCAGTCAC<br><br>ACACGTTCAGAGTTCTACAGTCCGACGATCGGGTTGGTGAAGTTGGTAGG	
Lrpap1_3	**GGGCTATGGCTCCACCACTGAGTTTGAAGAGCCCCGGGTG**<br><br>/5Phos/CAGTGGTGGAGCCATAGCCCTGGAATTCTCGGGTGCCAAGGAACTCCAGTCAC<br><br>ACACGTTCAGAGTTCTACAGTCCGACGATCCACCCGGGGCTCTTCAAACT	100
M6pr_2	**GGCTCGATCTTACTTGTCATATTTGCATCATTGGTTGCTG**<br><br>/5Phos/ATGACAAGTAAGATCGAGCCTGGAATTCTCGGGTGCCAAGGAACTCCAGTCAC<br><br>ACACGTTCAGAGTTCTACAGTCCGACGATCCAGCAACCAATGATGCAAAT	100
Pdia3_3	**ATAAAGAGCTGGGAGAAAAACTCAGCAAAGATCCAAATAT**<br><br>/5Phos/TTTTTCTCCCAGCTCTTTATTGGAATTCTCGGGTGCCAAGGAACTCCAGTCAC<br><br>ACACGTTCAGAGTTCTACAGTCCGACGATCATATTTGGATCTTTGCTGAG	100
Psmc4_2*	**TCAACTCCATCTGTCAGGAGAGTGGAATGTTGGCTGTCCG**<br><br>ACACGTTCAGAGTTCTACAGTCCGACGATCCGGACAGCCAACATTCCACT<br><br>/5Phos/CTCCTGACAGATGGAGTTGATGGAATTCTCGGGTGCCAAGGAACTCCAGTCAC	200
Ptafr_1	**CTCGGCCCTTTGATAGGAACTGAAGAGGGGCCAGGGTTGG**<br><br>/5Phos/GTTCCTATCAAAGGGCCGAGTGGAATTCTCGGGTGCCAAGGAACTCCAGTCAC<br><br>ACACGTTCAGAGTTCTACAGTCCGACGATCCCAACCCTGGCCCCTCTTCA	300
Rab5a_1	**TGAAATATTTATGGCAATAGCTAAAAAGCTGCCAAAGAAT**<br><br>/5Phos/CTATTGCCATAAATATTTCATGGAATTCTCGGGTGCCAAGGAACTCCAGTCAC<br><br>ACACGTTCAGAGTTCTACAGTCCGACGATCATTCTTTGGCAGCTTTTTAG	200
Sorl1_2	**AGCAGTTCAATGAAACCAGGGGCTATGAGATCCACATGTC**<br><br>/5Phos/CCTGGTTTCATTGAACTGCTTGGAATTCTCGGGTGCCAAGGAACTCCAGTCAC<br><br>ACACGTTCAGAGTTCTACAGTCCGACGATCGACATGTGGATCTCATAGCC	200
Sort1_2	**AAATATGTCTGTGGCGGAAGGTTCCTGGTGCACCGGTACT**<br><br>/5Phos/CTTCCGCCACAGACATATTTGGAATTCTCGGGTGCCAAGGAACTCCAGTCAC<br><br>ACACGTTCAGAGTTCTACAGTCCGACGATCAGTACCGGTGCACCAGGAAC	200
Tbp_2*	**ACCCACCAGCAGTTCAGTAGCTATGAGCCAGAATTATTTC**<br><br>ACACGTTCAGAGTTCTACAGTCCGACGATCGAAATAATTCTGGCTCATAG<br><br>/5Phos/CTACTGAACTGCTGGTGGGTTGGAATTCTCGGGTGCCAAGGAACTCCAGTCAC	200
Tgfbr1_1	**ACAGATGGCAGAGCTGTGAGGCCTTGAGAGTGATGGCTAA**<br><br>/5Phos/CTCACAGCTCTGCCATCTGTTGGAATTCTCGGGTGCCAAGGAACTCCAGTCAC<br><br>ACACGTTCAGAGTTCTACAGTCCGACGATCTTAGCCATCACTCTCAAGGC	100
Tnf-α_1	**TCGGTCCCCAAAGGGATGAGAAGTTCCCAAATGGCCTCCC**<br><br>ACACGTTCAGAGTTCTACAGTCCGACGATCGGGAGGCCATTTGGGAACTT<br><br>/5Phos/CTCATCCCTTTGGGGACCGATGGAATTCTCGGGTGCCAAGGAACTCCAGTCAC	300
Trem2_1	**CCAACTTCAGATCCTCACTGGACCCGGAGGTACGTGAGAG**<br><br>/5Phos/CAGTGAGGATCTGAAGTTGGTGGAATTCTCGGGTGCCAAGGAACTCCAGTCAC<br><br>ACACGTTCAGAGTTCTACAGTCCGACGATCCTCTCACGTACCTCCGGGTC	100
Tsg101_2	**CCCGCTTAGATCAAGAAGTAGCTGAAGTTGATAAAAACAT**<br><br>/5Phos/TACTTCTTGATCTAAGCGGGTGGAATTCTCGGGTGCCAAGGAACTCCAGTCAC<br><br>ACACGTTCAGAGTTCTACAGTCCGACGATCATGTTTTTATCAACTTCAGC	100

Ubqln2_2	**ATCTTTGTGCAGCCTGAAGGATCAGTGTAGTGACTCCAGG**<br>/5Phos/CCTTCAGGCTGCACAAAGATTGGAATTCTCGGGTGCCAAGGAACTCCAGTCAC<br>ACACGTTCAGAGTTCTACAGTCCGACGATCCCTGGAGTCACTACACTGAT	100
Vegf_3	**TTAAACGAACGTACTTGCAGATGTGACAAGCCAAGGCGGT**<br>ACACGTTCAGAGTTCTACAGTCCGACGATCACCGCCTTGGCTTGTCACAT<br>/5Phos/CTGCAAGTACGTTCGTTTAATGGAATTCTCGGGTGCCAAGGAACTCCAGTCAC	300

Supplementary Methods:

Complete list of R packages used: ggfortify[1]; ggplot2[2]; RColorBrewer[3], tidyr[4]; stringr[5]; plyr[6]; dplyr[7]; gplots[8]; stringi[9]; ggrepel[10]; tidyverse[11]; ComplexHeatmap[12]; dendextend[13]; circlize[14]; Rtsne[15]; rgl[16]; RUVSeq[17]; DESeq2[18]; VennDiagram[19].

HCS Studio Cell Analysis Software, HealthCellProfiling protocol

First, the images were pre-processed; channel 1 had background removed using low pass filter value at 128, and channel 2 had background removed using low pass filter value at 255. Second, primary objects (microglia) were identified with smoothed channel 1 image by thresholding method with isodata set at -0.308. The validation of primary objects (microglia) was done on background removed channel 1 image by selecting objects with area between 30.25 and 168.801 and object average intensity between 0 and 6847.97. This way, objects with area too small (dust) or too big (debris), and cells with high Hoechst nuclear staining indicative of cell death were removed. The resultant valid object count was used for cell number analysis. To select the area for BMV109 signal quantification, a mask was applied on background removed channel 2 images with Circ value at 10 to draw non-overlapping areas around each Hoechst-stained nucleus, and total intensity set between 0 and 1,770,021 to exclude the few bright outliers. Third, for quantification of the signal, the target thresholding method with isodata set at -0.5 inside the area outlined above (Circ value = 10) was used on the background removed channel 2; the per-cell and per-well (mean target average intensity, channel 2) was used for analysis. Signal in the 650 nm channel from blank wells containing microglia that were only stained with Hoechst was subtracted from all wells containing BMV109 probe.

1 Tang, Y., Horikoshi, M. & Li, W. ggfortify: Unified Interface to Visualize Statistical Result of Popular R Packages. *The R Journal* **8** (2016).
2 Wickham, H. *ggplot2: Elegant Graphics for Data Analysis*. (Springer-Verlag New York, 2016).
3 Neuwirth, E. *RColorBrewer: ColorBrewer Palettes*. (2014).
4 Wickham, H. & Henry, L. tidyr: Easily Tidy Data with 'spread()' and 'gather()' Functions. (2018).
5 Wickham, H. *stringr: Simple, Consistent Wrappers for Common String Operations*. (2018).

6 Wickham, H. The Split-Apply-Combine Strategy for Data Analysis. *Journal of Statistical Software* **40**, 1-29 (2011).
7 Wickham, H., Francois, R., Henry, L. & Muller, K. dplyr: A Grammar of Data Manipulation. (2018).
8 Warnes, G. R. *et al.* gplots: Various R Programming Tools for Plotting Data. (2016).
9 Gagolewski, M. R package stringi: Character string processing facilities. (2018).
10 Slowikowski, K. ggrepel: Automatically Position Non-Overlapping Text Labels with 'ggplot2'. (2018).
11 Wickham, H. tidyverse: Easily Install and Load the 'Tidyverse'. (2017).
12 Gu, Z., Eils, R. & Schlesner, M. Complex heatmaps reveal patterns and correlations in multidimensional genomic data. *Bioinformatics* (2016).
13 Galili, T. dendextend: an R package for visualizing, adjusting, and comparing trees of hierarchical clustering. *Bioinformatics*, doi:10.1093/bioinformatics/btv428 (2015).
14 Gu, Z., Gu, L., Eils, R., Schlesner, M. & Brors, B. circlize implements and enhances circular visualization in R. *Bioinformatics* **30**, 2811-2812 (2014).
15 Krijthe, J. H. {Rtsne}: T-Distributed Stochastic Neighbor Embedding using Barnes-Hut Implementation. (2015).
16 Adler, D. *et al.* rgl: 3D Visualization Using OpenGL. (2019).
17 Risso, D., Ngai, J., Speed, T. P. & Dudoit, S. Normalization of RNA-seq data using factor analysis of control genes or samples. *Nat Biotechnol* **32**, 896-902, doi:10.1038/nbt.2931 (2014).
18 Love, M. I., Huber, W. & Anders, S. Moderated estimation of fold change and dispersion for RNA-seq data with DESeq2. *Genome Biol* **15**, 550, doi:10.1186/s13059-014-0550-8 (2014).
19 Chen, H. VennDiagram: Generate High-Resolution Venn and Euler Plots. (2018).

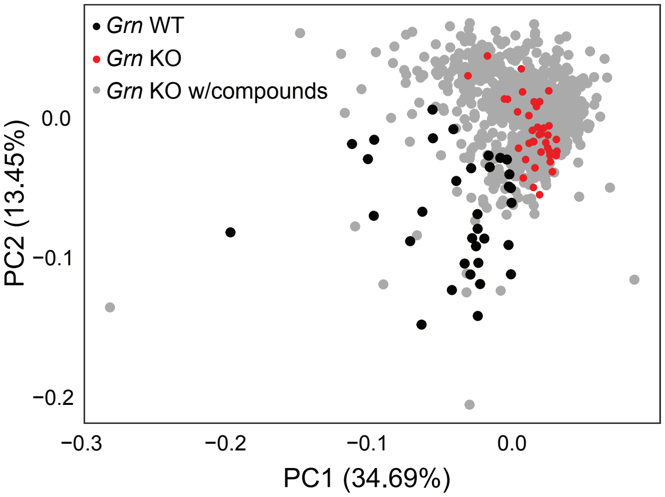

Supplementary Figure S1. PCA plot representing mRNA expression of 11 signature genes of *Grn* WT, *Grn* KO, and compound-treated *Grn* KO microglia cells.

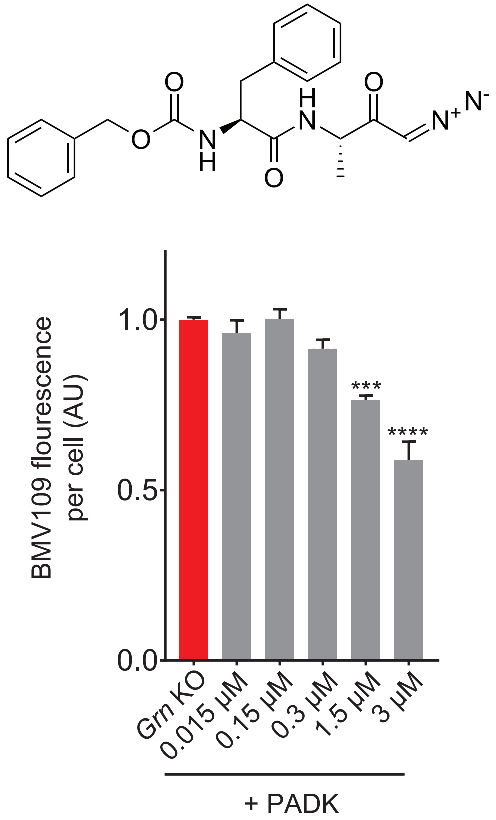

Supplementary Figure S2. Cathepsin inhibitor PADK treatment lowers BMV109 signal in *Grn* KO microglia. BMV109 signal (mean ± SEM) is from 3 independent experiments, n=8 for *Grn* KO, n = 3-6 for each concentration of PADK added to *Grn* KO cells.

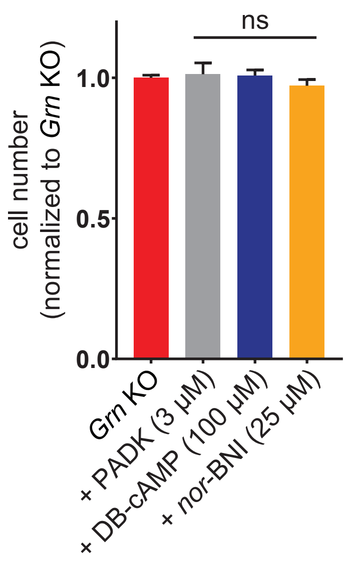

Supplementary Figure S3. Cell viability with addition of transcriptional correctors to *Grn* KO microglia cells. BMV109 signal (mean ± SEM) data are pooled from 5 independent experiments, n=24 for *Grn* KO, n=5-8 for each compounds added to *Grn* KO cells.

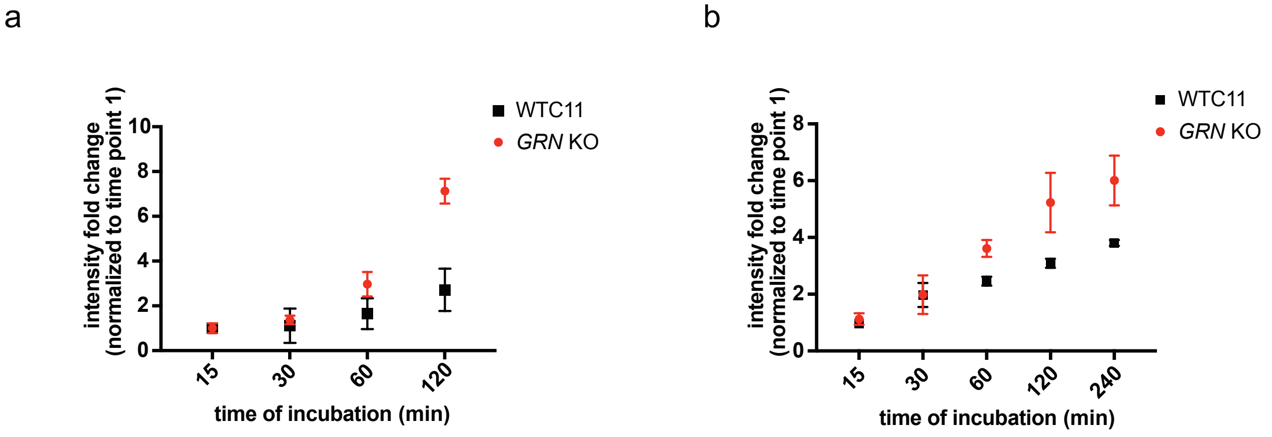

Supplementary Figure S4. BrdU incorporation into WTC11 and *GRN* KO iPSCs. (a) BrdU incorporation on day 3 of plating (40% confluency) and (b) day 4 of plating (80% confluency). n = 3 for each experiment.

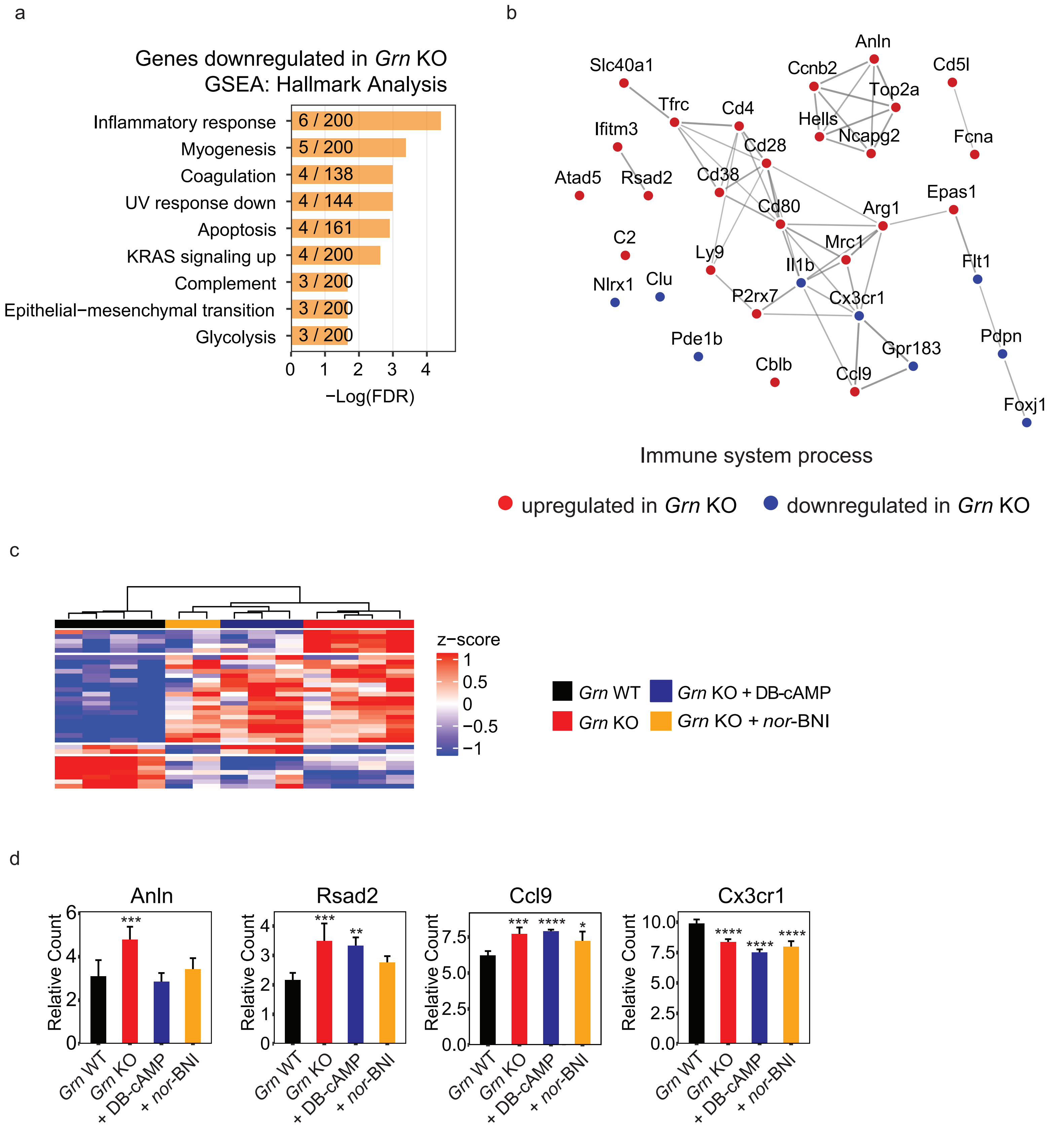

Supplementary Figure S5. Effect of compounds on immune system process in *Grn* KO microglia cells. (a) GSEA Hallmark analysis of downregulated genes in *Grn* KO microglia. (b) Cytoscape network of STRING-classified immune system process genes dysregulated in *Grn* KO vs WT microglia. (c) Heatmap and unbiased Ward column clustering of immune system process genes demonstrate effect of compound addition to *Grn* KO cells. (d) mRNA expression of individual genes. n=2-4; * p-value ≤ 0.05, ** p-value ≤ 0.01, *** p-value ≤ 0.001, **** p-value ≤ 0.0001.

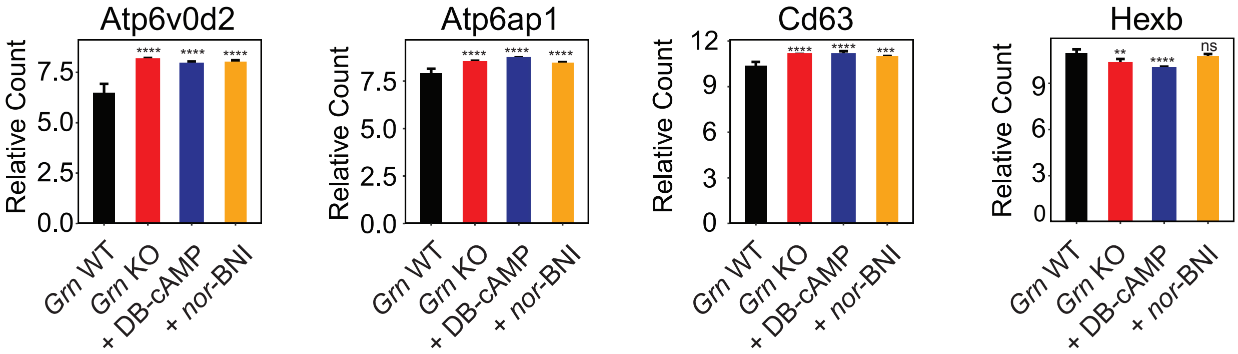

Supplementary Figure S6. mRNA expression levels of selected lysosomal genes. n=2-4 ** p-value ≤ 0.01, **** p-value ≤ 0.0001. Log2FC changes (KO vs WT): *Atp6ap1*, 1.61; *Atp6v0d2*, 0.52; *Cd63*, 0.69; *Hexb*, -0.68.

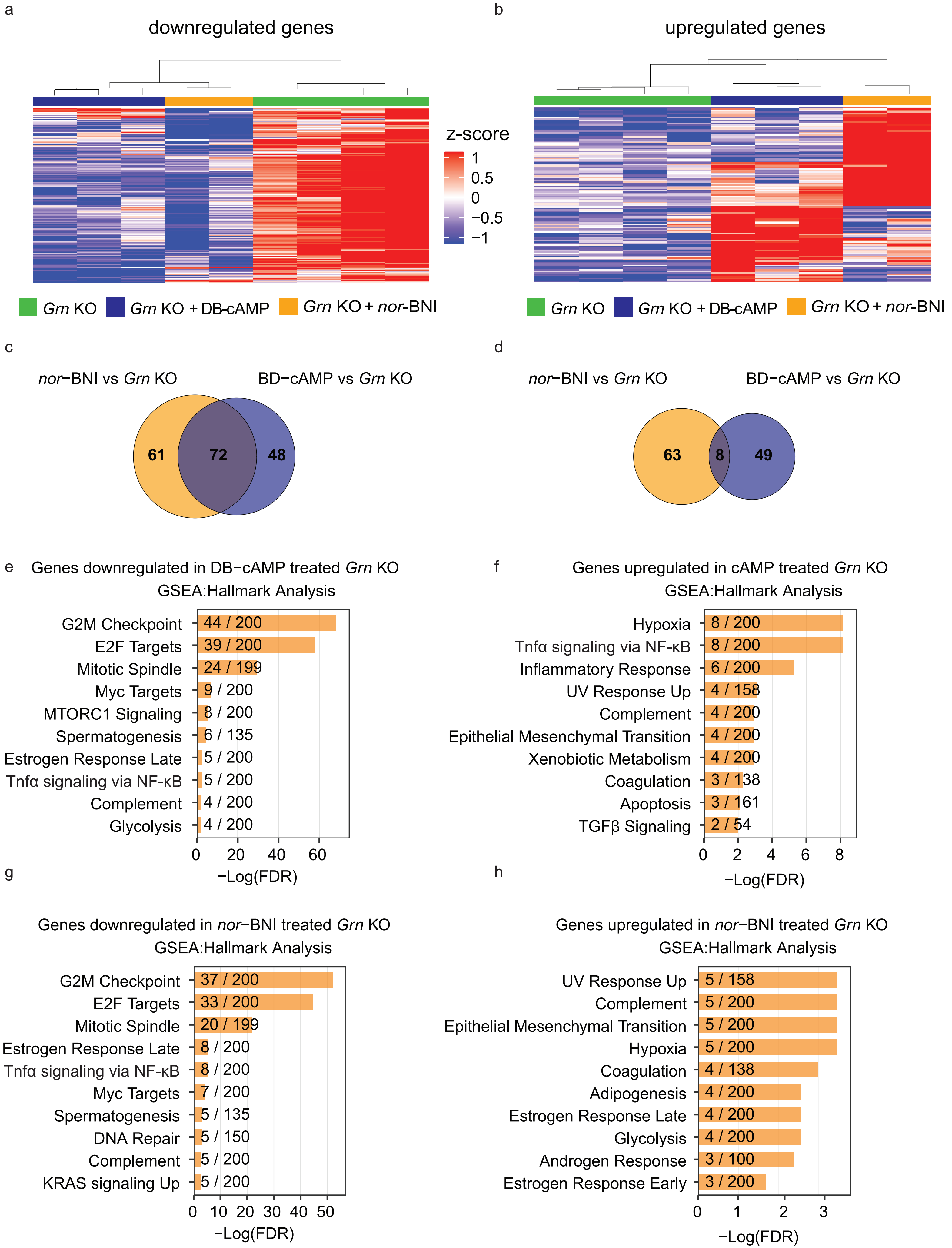
a
downregulated genes
z-score
1
0.5
0
−0.5
−1
Grn KO
Grn KO + DB-cAMP
Grn KO + nor-BNI
b
upregulated genes
Grn KO
Grn KO + DB-cAMP
Grn KO + nor-BNI
c
nor−BNI vs Grn KO
BD−cAMP vs Grn KO
61
72
48
d
nor−BNI vs Grn KO
BD−cAMP vs Grn KO
63
8
49
e
Genes downregulated in DB−cAMP treated Grn KO
GSEA:Hallmark Analysis
G2M Checkpoint
44 / 200
E2F Targets
39 / 200
Mitotic Spindle
24 / 199
Myc Targets
9 / 200
MTORC1 Signaling
8 / 200
Spermatogenesis
6 / 135
Estrogen Response Late
5 / 200
Tnfα signaling via NF-κB
5 / 200
Complement
4 / 200
Glycolysis
4 / 200
0
20
40
60
−Log(FDR)
f
Genes upregulated in cAMP treated Grn KO
GSEA:Hallmark Analysis
Hypoxia
8 / 200
Tnfα signaling via NF-κB
8 / 200
Inflammatory Response
6 / 200
UV Response Up
4 / 158
Complement
4 / 200
Epithelial Mesenchymal Transition
4 / 200
Xenobiotic Metabolism
4 / 200
Coagulation
3 / 138
Apoptosis
3 / 161
TGFβ Signaling
2 / 54
0
2
4
6
8
−Log(FDR)
g
Genes downregulated in nor−BNI treated Grn KO
GSEA:Hallmark Analysis
G2M Checkpoint
37 / 200
E2F Targets
33 / 200
Mitotic Spindle
20 / 199
Estrogen Response Late
8 / 200
Tnfα signaling via NF-κB
8 / 200
Myc Targets
7 / 200
Spermatogenesis
5 / 135
DNA Repair
5 / 150
Complement
5 / 200
KRAS signaling Up
5 / 200
0
10
20
30
40
50
−Log(FDR)
h
Genes upregulated in nor−BNI treated Grn KO
GSEA:Hallmark Analysis
UV Response Up
5 / 158
Complement
5 / 200
Epithelial Mesenchymal Transition
5 / 200
Hypoxia
5 / 200
Coagulation
4 / 138
Adipogenesis
4 / 200
Estrogen Response Late
4 / 200
Glycolysis
4 / 200
Androgen Response
3 / 100
Estrogen Response Early
3 / 200
0
1
2
3
−Log(FDR)

Supplementary Figure S7. Effect of compounds in *Grn* KO microglia cells. (a), (b) Heatmaps and unbiased column clustering (Ward's method with Euclidean distance) of all (a) downregulated and (b) upregulated genes in compound-treated cells compared to untreated *Grn* KO controls. (c), (d) Venn diagrams comparing differentially expressed genes that were (c) downregulated and (d) upregulated between DB-cAMP and *nor*-BNI treated *Grn* KO cells compared to untreated *Grn* KO cells. DE genes were calculated using log2FC difference of ≥1 or ≤-1 and p-value ≤0.005. (e)-(h) GSEA Hallmark analysis of differentially expressed genes in DB-cAMP and *nor*-BNI treated *Grn* KO cells compared to untreated *Grn* KO cells: (e) downregulated in DB-cAMP-, (f) upregulated in DB-cAMP-, (g) dowrnegulated in *nor*-BNI-, and (h) upregulated in *nor*-BNI- treated *Grn* KO cells compared to untreated *Grn* KO cells.
